# In Vivo Investigation of Polymer-Ceramic PCL/HA and PCL/β-TCP 3D Composite Scaffolds and Electrical Stimulation for Bone Regeneration

**DOI:** 10.3390/polym14010065

**Published:** 2021-12-25

**Authors:** Júlia Venturini Helaehil, Carina Basqueira Lourenço, Boyang Huang, Luiza Venturini Helaehil, Isaque Xavier de Camargo, Gabriela Bortolança Chiarotto, Milton Santamaria-Jr, Paulo Bártolo, Guilherme Ferreira Caetano

**Affiliations:** 1 Graduate Program in Biomedical Sciences, University Center of Hermínio Ometto Foundation, FHO, Araras 13607-339, SP, Brazil; juliaventurini.h@gmail.com (J.V.H.); carinabasqueira@fho.edu.br (C.B.L.); luizahelaehil@gmail.com (L.V.H.); gabichiarotto@fho.edu.br (G.B.C.); santamariajr@fho.edu.br (M.S.-J.); 2 Department of Mechanical, Aerospace and Civil Engineering, School of Engineering, University of Manchester, Manchester M13 9PL, UK; boyang.huang@manchester.ac.uk; 3 Graduate Program of Orthodontics, University Center of Hermínio Ometto Foundation, FHO, Araras 13607-339, SP, Brazil; isaque__22@hotmail.com; 4 Singapore Centre for 3D Printing, School of Mechanical and Aerospace Engineering, Nanyang Technological University, Jurong West 639798, Singapore

**Keywords:** hydroxyapatite, β-tricalcium phosphate, additive manufacturing, electrical stimulation, bone regeneration

## Abstract

Critical bone defects are a major clinical challenge in reconstructive bone surgery. Polycaprolactone (PCL) mixed with bioceramics, such as hydroxyapatite (HA) and tricalcium phosphate (TCP), create composite scaffolds with improved biological recognition and bioactivity. Electrical stimulation (ES) aims to compensate the compromised endogenous electrical signals and to stimulate cell proliferation and differentiation. We investigated the effects of composite scaffolds (PCL with HA; and PCL with β-TCP) and the use of ES on critical bone defects in Wistar rats using eight experimental groups: untreated, ES, PCL, PCL/ES, HA, HA/ES, TCP, and TCP/ES. The investigation was based on histomorphometry, immunohistochemistry, and gene expression analysis. The vascular area was greater in the HA/ES group on days 30 and 60. Tissue mineralization was greater in the HA, HA/ES, and TCP groups at day 30, and TCP/ES at day 60. Bmp-2 gene expression was higher in the HA, TCP, and TCP/ES groups at day 30, and in the TCP/ES and PCL/ES groups at day 60. Runx-2, Osterix, and Osteopontin gene expression were also higher in the TCP/ES group at day 60. These results suggest that scaffolds printed with PCL and TCP, when paired with electrical therapy application, improve bone regeneration.

## 1. Introduction

Bone tissue has a high capacity for repair after trauma or injury. However, this potential becomes compromised in large bone defects, requiring an effective approach that allows bone growth. Autologous bone grafting remains the gold standard in bone repair; however, it is associated with several clinical setbacks, such as limited availability of healthy bone, high costs, mandatory secondary surgery, morbidity at the bone harvesting site, and long-term pain problems [1,2,3].

A rapidly arising method in this field uses additive manufacturing to regenerate extensive bone defects by developing three-dimensional porous support structures (bone scaffolds) that contribute to new tissue formation based on their osteoconductive capacity [4,5]. The use of synthetic biomaterials to act as bone grafts, thereby promoting successful bone regeneration, has been widely investigated [6,7]. The use of bioceramics based on calcium phosphate salts, such as hydroxyapatite (HA) and tricalcium phosphate (β-TCP), is widely accepted for bone tissue engineering as both are biocompatible with, and can provide, chemical similarity to the inorganic components of natural bone tissue [8,9,10,11,12]. The combination of polycaprolactone (PCL) with bioceramics to create composite scaffolds has been reported to improve the performance and function by increasing biological recognition and bioactivity [12,13]. Bioceramics composed of calcium phosphate are considered a class of bioactive materials that are widely used for bone tissue repair [7,8,14]. However, not all of them are able to promote the same repair effect in vivo, as most are osteoconductive; only some of these materials are osteoinductive [15]. Our research group reported the fabrication process of composite scaffolds and their mechanical and biological properties in vitro [7].

Electrical stimulation (ES) is a low-intensity electrical current that is a non-invasive clinical therapy used to enhance the bone regeneration process, complementary to scaffold grafting. Its application is aimed at compensating for the lack of electrical signals after bone loss and to promote cell migration, proliferation, and differentiation [2,16,17]. The application of electrical stimulation allows the transmission of ionic signals, thus promoting osteogenic differentiation, as well as compensating for compromised endogenous electrical signals and osteointegration [18,19]. Considering the use of ES and composite scaffolds produced with an appropriate concentration of bioceramics, similar to the calcium phosphate nature of native bone tissue, this study used additive manufacturing technology combined with synthetic bioceramics to produce 3D porous scaffolds to mimic the native bone. The in vivo performance of the produced scaffolds, with/without ES, were comprehensively assessed using histomorphometry, immunohistochemistry, and gene expression. This study provides a preliminary study, and a potential clinical approach, to treat large bone defects using ES and 3D bioceramic scaffolds.

## 2. Experimental Methods

### 2.1. Scaffold Fabrication

**Figure 1:**
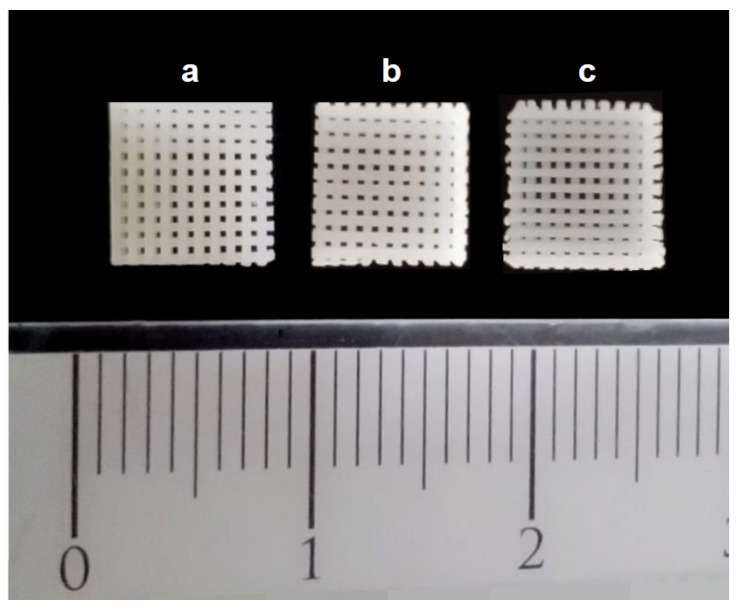
Three-dimensional scaffolds produced by additive manufacturing (5 mm × 5 mm × 2 mm) before implantation into the bone defects. (**a**) PCL scaffold, (**b**) PCL with HA scaffold, and (**c**) PCL with TCP scaffold (unit: centimeter).

PCL/HA and PCL/TCP scaffolds were fabricated as previously reported [7]. In brief, melt blending was used to produce composite materials. PCL pellets (Perstorp Caprolactones, Cheshire, UK) were heated up to 90 °C and mixed with HA nanoparticles (purisis ≥ 97%, below 200 nm) (Sigma-Aldrich, St. Louis, MO, USA) or β-TCP microparticles (purisis ≥ 98%) (Sigma-Aldrich, St. Louis, MO, USA) according to the designed scaffold weight ratios (Table 1). Then, they were manually mixed for at least 20 min to obtain homogeneous mixtures. The prepared composite materials were 3D printed with a screw-assisted 3D Discovery (REGENHU, Villaz-Saint-Pierre, Switzerland) using the following configuration: 0/90° lay-down pattern, 90 °C melting temperature, 20 mm/s feed rate, and 12 rpm screw rotational velocity. The scaffolds were printed with a pore size of 350 µm, layer thickness of 270 µm, and height of 2.5 mm, using a nozzle with a 330 µm diameter. The fabricated scaffolds were sterilized in 70% ethanol for 4 h, rinsed with sterile saline solution, and dried overnight in a sterile fume hood. The scaffolds were cut off and adjusted to the critical size of bone lesion during the surgical procedure. The printed scaffolds are shown in Figure 1.

### 2.2. In-Vivo Study

#### Animals

Wistar rats were obtained from the Animal Experimentation Center at the University Center of Hermínio Ometto Foundation (Brazil) and were housed in collective polycarbonate boxes, with food and water *ad libitum*. The animals were randomly divided into eight experimental groups, as listed in Table 1. Each group was further divided into another three subgroups to consider three experimental periods: 30, 60, and 120 d (*n* = 8 animals/group/experimental period).

All surgical and experimental procedures were performed according to experimental standards and biodiversity rights (NIH Publication 80–23, revised 1996 and Arouca Law-11, 794, 2008) and approved by the ethical principles in animal research adopted by Hermínio Ometto Foundation’s Ethics Committee on Animal Use (CEUA 075/2017). During the experimental periods, the animals were healthy and adapted to the treatment without stress.

### 2.3. Surgical Protocol

**Figure 2:**
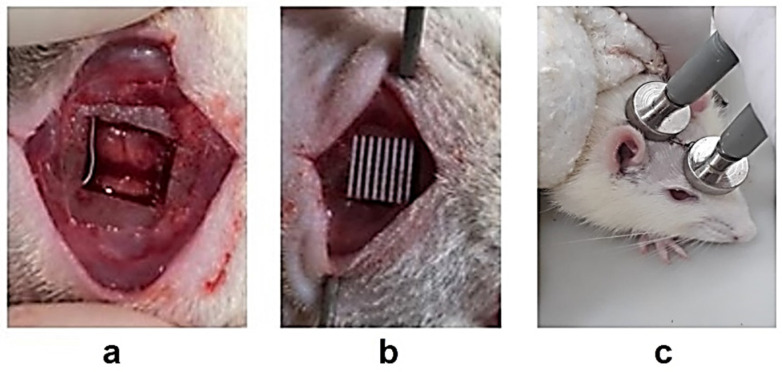
Scaffold implantation and electrical stimulation therapy. (**a**) Critical-sized bone defect; (**b**) implanted scaffold into the bone defect; (**c**) two electrode probes in contact with the animal for electrical stimulation.

The animals were anesthetized by the intraperitoneal administration of a mixture of ketamine hydrochloride (30 mg/kg) and xylazine hydrochloride (10 mg/kg), followed by the trichotomy of the occipital region of the animals. A critical-sized bone defect with dimensions of 5 mm × 5 mm, was created in the center of the calvary bone (Figure 2a) under constant irrigation with physiological solution (NaCl 0.9%) using an Osteo I tip (Piezo Helse, Helse Dental Technology, Santa Rosa do Viterbo, SP, Brazil) coupled with a dental ultrasound handpiece (Olsen, Palhoça, SC, Brazil).

The scaffolds were sterilized in 70% ethanol before being precisely fitted to the bone defect with no need for clamping or physical fixation (Figure 2b). After scaffold implantation, the wounds were sutured with nylon 5-0 sutures (Shalon Medical, Goiânia, Brazil), followed by intraperitoneal and oral analgesic treatments using tramadol hydrochloride (1 mg/kg) and dipyrone (50 mg/kg), respectively, for 72 h. Additionally, the animals were monitored by the researchers.

### 2.4. Electrical Stimulation and Post Treatment

Electrical stimulation (ES) was performed using a low-intensity transcutaneous electrical stimulator (Physeotonus microcurrent, BIOSET, Indústria de Tecnologia Electrônica Ltd.a, Rio Claro, São Paulo, Brazil). Two conductive metal electrode probes were placed gently in contact with the animal’s head, around the bone defect (Figure 2c), for 5 min at 10 μA (galvanic electrical current), twice a week, throughout the three experimental periods. The efficacy of the ES protocol, and its clinical reasoning, have been discussed previously [20,21,22].

After the experimental periods of 30, 60, and 120 d after surgery, the animals were euthanized by cervical dislocation under deep anesthesia. Samples were collected considering the area covering the entire bone defect/scaffold (the new tissue formation) and approximately 2 mm of the bone edges. From the total samples of each group, three were collected for histomorphometric and immunohistochemical (*n* = 3/group/experimental period) evaluation and then immediately fixed in 10% formaldehyde for 48 h; five samples were immediately frozen at −80 °C in 2 mL plastic tubes for molecular evaluation (*n* = 5/group/experimental period).

### 2.5. Histomorphometry

After fixation, the samples were transferred to a 50% buffered formic acid decalcifying solution (Morse’s decalcifying) for 45 d. The solution was changed three times per week. After demineralization, the samples were rinsed in running water, dehydrated in ethanol concentration, diaphanized with xylol, embedded in paraffin and 4.0 μm thick cross-sectioned, mounted on glass slides, and stained with hematoxylin-eosin (HE) or Masson’s Trichrome (MT) for histomorphometric evaluation. The histological images were captured using a Leica DM2000 microscope (Leica Microsystems, Wetzlar, Germany). The samples stained with HE were captured at 100× magnification, and the samples stained with MT were captured at 200× magnification for histomorphometric evaluation (number of blood vessels, vascular area, osteoid/collagen tissue, and mineralized tissue). Ten images of each bone sample (each animal) were analyzed using ImageJ software.

### 2.6. Immunohistochemistry 

Cross-sections of 4.0 μm thickness were also used for immunohistochemistry. The samples were incubated with the primary antibodies, anti-RANKL (sc-377079, 1:200; Santa Cruz Biotechnology, Dallas, TX, USA) and anti-OPG (sc-390518, 1:200; Santa Cruz Biotechnology, Dallas, TX, USA), to evaluate bone resorption and bone remodeling. The secondary antibodies and antibody detection reaction (NovolinkTM Max Polymer Detection System) were performed according to the manufacturer’s recommended protocol (Leica Biosystems, Buffalo Grove, IL, USA). Diaminobenzidine (DAB) was used as a substrate chromogen to reveal the specific markings, followed by hematoxylin counterstaining. Ten images of each bone sample were captured at 400× using a Leica DM2000 microscope (Leica Microsystems, Wetzlar, Germany) and quantified using ImageJ software.

### 2.7. Quantitative Polymerase Chain Reaction (qPCR) 

Gene expression analysis was performed on defect tissues collected 30 and 60 d post-surgery, and the samples were macerated with liquid nitrogen. Total RNA was isolated using TRIzol^TM^ reagent (Invitrogen, Waltham, MA, USA), following the manufacturer’s instructions. Cell lysis was performed using a homogenizer (Polytron System PT 1200E, Kinematica AG, Malters, Switzerland). The quantity and quality of RNA samples were measured using a spectrophotometer at the ratios A260/230 and A260/280. The integrity of the RNA was verified by electrophoresis on a 1% agarose gel. The cDNA was synthesized from 1.5 µg of total RNA using the high-capacity kit (Thermo Fisher Scientific, code 4374966) according to the manufacturer’s instructions. For qPCR reactions, the TaqMan assays *Gapdh* (Rn01775763_g1), *Vegf* (Rn01511602_m1), *Runx2* (Rn01512298_m1), *Osterix* (Sp7) (Rn02769744_s1), *Bmp-2* (Rn00567818_m1), *Bmp-7* (Rn01528889_m1), and *osteopontin* (Rn00681031_m1) were purchased from Applied Biosystems, and the reactions were performed in triplicate with TaqMan Gene Expression Master Mix (Applied Biosystems, Waltham, MA, USA). The entire qPCR procedure was performed on the QuantStudio 3 Real-Time PCR Systems instrumentation platform (Thermo Fisher, Waltham, MA, USA), and the thermal cycling conditions used were 95 °C for 10 min, followed by 45 cycles at 95 °C for 15 s and 60 °C for 1 min. All expression levels were normalized to GAPDH and validated using the BestKeeper software, then used as the normalizer. The PCL group was used as a calibrator. The results were calculated using the 2^−ΔΔCt^ method [23].

### 2.8. Statistical Analysis

All experimental data are presented as the mean ± standard error of the mean. Data were analyzed using GraphPad Prism 8 software (GraphPad Software, San Diego, CA, USA) and verified using the normality test. One-way ANOVA with Tukey’s post-hoc test was applied for parametric data, while the Kruskal–Wallis test with Dunn post-hoc, was applied for non-parametric data. Significance levels were set at: * *p* < 0.05; ** *p* < 0.01; *** *p* < 0.001.

## 3. Results

### 3.1. Histomorphometry

**Figure 3:**
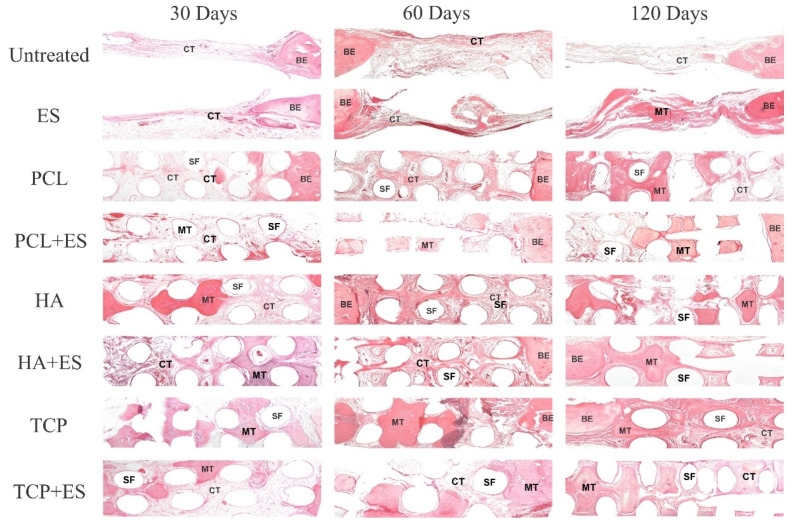
Hematoxylin-eosin (HE) cross-section images of bone tissue regeneration. Tissue formation is shown among the scaffold filaments and into the bone defect after 30, 60, and 120 d of the bone regeneration process. The image shows the bone edge (BE), connective tissue (CT), mineralized tissue (MT), and the scaffold filaments (SF).

Figure 3 show the representative histological images of the implanted scaffold and tissue stained with HE. The observed bone edge (BE), connective tissue (CT), mineralized tissue (MT), and the scaffold filaments (SF) are labeled accordingly. The presence of CT was more evident in the untreated and ES groups over the experimental period. However, MT was more evident in the groups that received the scaffolds, especially in the HA and HA/ES groups at day 30, and the TCP and TCP/ES groups after 60 and 120 d. As the bone defect is considered critical, the regeneration process, followed by the mineralization of the osteoid/connective tissue, was not observed in the untreated and Es groups where scaffolds were not used for bone grafting.

**Figure 4:**
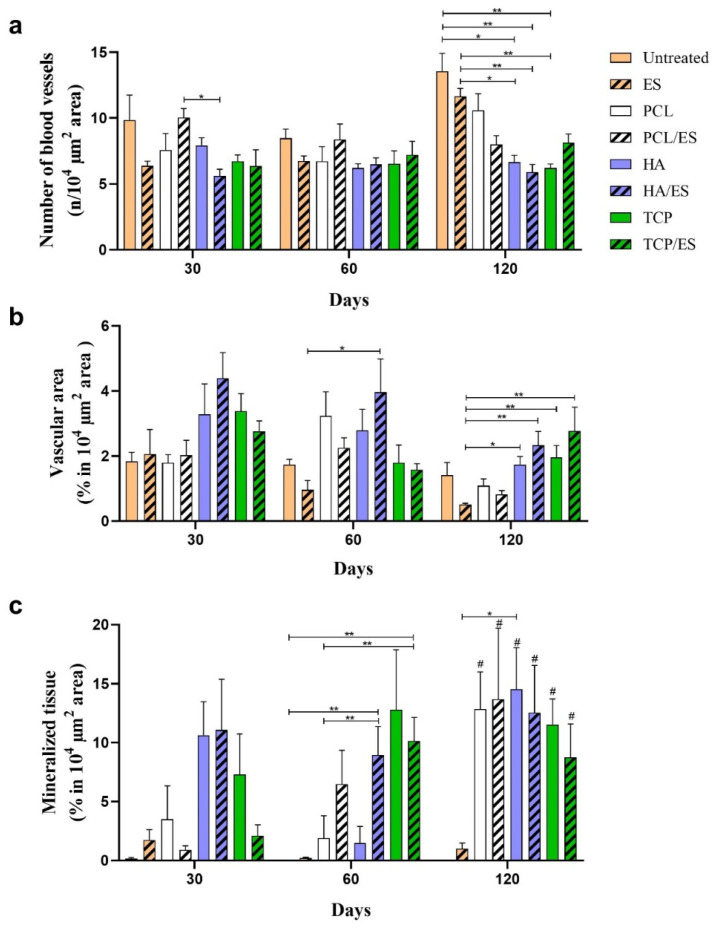
Histological quantification. (**a**) Number of blood vessels presented in 10^4^ µm^2^ area of each image of the bone defect; (**b**) vascular area (percentage) in 10^4^ µm^2^ area of the bone defect; (**c**) mineralized tissue area (percentage) in 10^4^ µm^2^ area of the bone defect. * (*p* < 0.05); ** (*p* < 0.01); # (statistical difference to untreated group).

Figure 4a present the quantification of blood vessels in a 10^4^ µm^2^ histological image area for all histological images stained with Masson’s trichrome. On day 30, the PCL/ES group presented an overall higher number of blood vessels compared to the other groups. Although the untreated group also presented values similar to the PCL/ES group, there was no statistically significant difference compared to the other groups. No difference was observed on day 60 in any of the samples. However, on day 120, the untreated and ES groups showed overall higher blood vessel numbers compared to the HA, HA/ES, and TCP groups. These results indicate that the addition of HA and TCP particles had no significant impact on the vascularization process.

Figure 4b show the percentage of the vascular area considering the blood vessels in the histological images. The overall vascular area of all samples decreased from day 30 to day 120 of the experimental period. This may be caused by the formation of more mature bone. On day 30, the results showed that groups containing HA and TCP particles showed an increased vascular area compared to the non-ceramic groups, especially the HA/ES group, which showed the largest vascular area compared to the other groups. After 60 d, the HA/ES group had a higher percentage of vascular area than the ES group. A similar trend was also observed at day 120, and it can be seen that the HA/ES and TCP/ES groups presented a statistically higher percentage of vascular area than the ES group. These results indicate that the addition of HA and TCP particles enhances vascularization during bone tissue regeneration. The use of ES can be seen to have slightly increased the vascular area; however, no statistically significant difference was observed between the groups with and without ES.

Figure 4c show the formation of mineralized tissue replacing the connective/osteoid tissue. On day 30, the HA, HA/ES, and TCP groups showed a higher percentage of mineralized tissue compared to the other groups. After 60 d, the HA/ES and TCP/ES groups presented a statistically higher percentage of mineralized tissue compared to the PCL and untreated groups. Moreover, the PCL/ES and TCP groups also presented evident mineralized tissue formation compared to the untreated, ES, and PCL groups. After 120 d, all scaffold-treated groups showed higher mineralized tissue than the untreated and ES groups. Both the untreated and ES groups were unable to mineralize connective tissue over the experimental period. The addition of HA and TCP particles enhanced mineralization. However, although ES seems to have an influence on the vascular area (angiogenesis), no considerable effect was observed on mineralization.

### 3.2. Osteogenic Gene Expression

**Figure 5:**
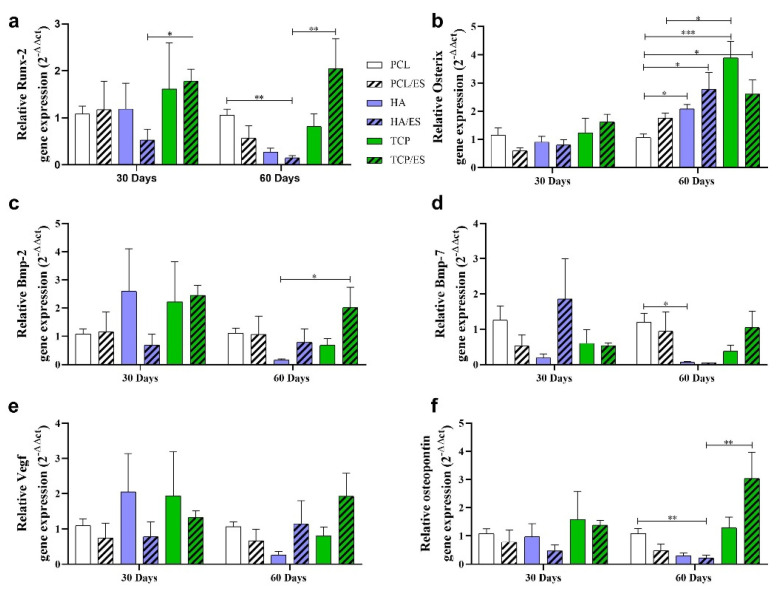
Gene expression showing the relative fold change for (**a**) Runx-2; (**b**) Osterix; (**c**) Bmp-2; (**d**) Bmp-7; (**e**) Vegf; and (**f**) Osteopontin. * (*p* < 0.05); ** (*p* < 0.01); *** (*p* < 0.001).

To further investigate the effects of ceramic scaffolds and ES on the bone regeneration process, osteogenic gene expression was examined. Based on the histological analysis of mineralized tissue, non-expressive tissue formation occurred in both the untreated and ES groups. Therefore, these two groups were not considered in this investigation. Figure 5a show the relative *Runx-2* gene expression. On day 30, the TCP/ES group showed a statistically significant difference compared to the HA/ES group. On day 60, both the PCL and TCP/ES groups showed higher expression of *Runx-2* compared to the HA/ES group. Moreover, the TCP/ES group presented a 2-fold change over PCL and even higher than the other groups.

Figure 5b show the relative expression of Osterix. All groups displayed a similarly low expression after 30 d. However, on day 60, in the groups with HA and TCP particles, the Osterix gene expression increased, and the TCP group showed statistically higher relative expression compared to the PCL (3.5-fold change) and PCL/ES (2-fold change) groups. It can also be observed that the TCP/ES, HA/ES, and HA groups presented higher expression (2.5-fold change) than the PCL group.

Figure 5c show the relative *Bmp-2* gene expression. On day 30, the HA, TCP, and TCP/ES groups showed higher expression, even without significant evidence. On day 60, the TCP/ES group presented higher expression compared to the HA/ES group and twice as high as the PCL group.

Figure 5d show the relative *Bmp-7* gene expression. The HA/ES group showed higher expression than the other groups at day 30. On day 60, HA and HA/ES showed significantly lower *Bmp-7* expression than the other groups.

Figure 5e show that on day 30, the HA and TCP groups presented higher expression of Vgef than the other groups; however, there was no significant difference. The same was observed on day 60 for the HA/ES and TCP/ES groups.

Figure 5f show the relative expression of OPN. On day 30, all groups showed similar expression. However, on day 60, the TCP/ES and PCL groups showed higher expression than HA/ES. For TCP/ES, expression was three times higher than the PCL.

### 3.3. Bone Remodelling 

**Figure 6:**
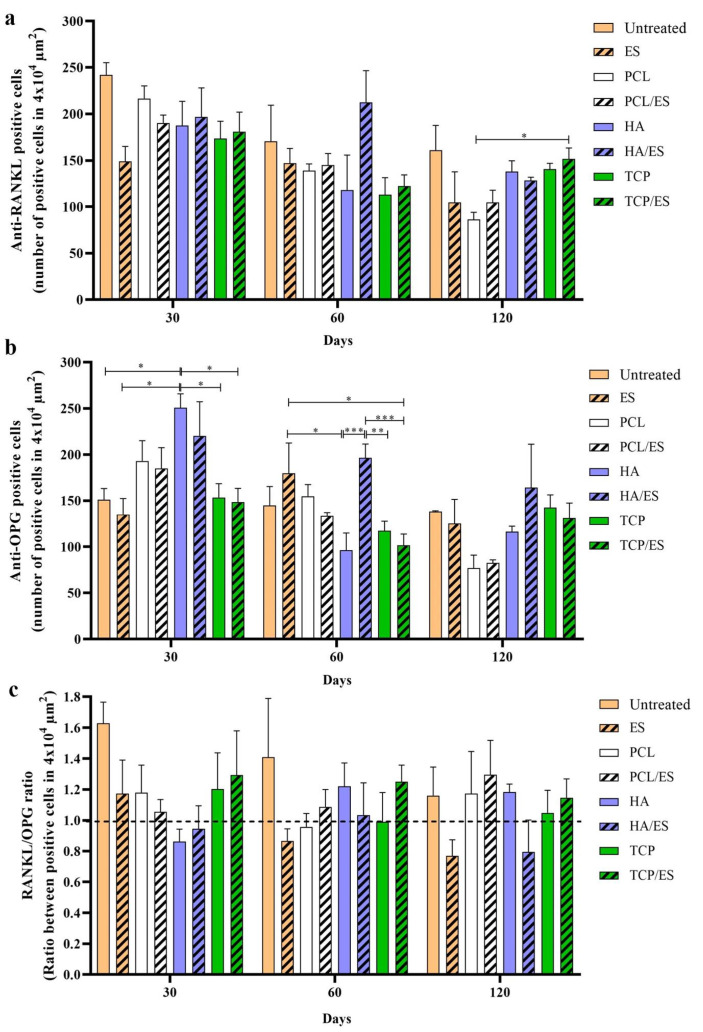
Evaluation of tissue remodeling by immunohistochemistry. (**a**) Quantification of anti-RANKL positive cells in 4 × 10^4^ μm^2^ area in the images; (**b**) quantification of anti-OPG positive cells in 4 × 10^4^ μm^2^ area in the images; (**c**) ratio of anti-RANKL and anti-OPG positive cells—values above 1.0 suggest a trend toward bone resorption, while values below 1.0 suggest tissue formation. * (*p* < 0.05); ** (*p* < 0.01); *** (*p* < 0.001).

The investigation of tissue remodeling was assessed by immunolabeling using specific antibodies. The results shown in Figure 6 consider the number of positively immunoreactive cells. Figure 6a show the number of RANKL-positive cells. The PCL, HA, HA/ES, TCP, and TCP/ES groups presented similar numbers of positive cells (around 180–200 positive cells) on day 30. The untreated group had almost 250 positive cells, ES group 150 cells, and PCL 230 cells. After 60 d, the number of positive cells declined for most of the groups, except for the HA/ES group, which continued with approximately 220 positive cells, and the ES groups with approximately 150 cells (no statistical difference). After 120 d, the TCP/ES group presented significantly more cells than the PCL group.

Figure 6b show the number of OPG-positive cells. After 30 d, the HA group presented a higher number of positive cells (around 250 cells) with evidence of difference compared to the untreated (150), ES (140), TCP (150), and TCP/ES (150) groups. After 60 d, the HA/ES presented a higher cell number (200) compared to the TCP (120), TCP/ES (100) groups, and the HA group (100). The ES group also presented higher numbers (180) than the HA and TCP/ES groups. After 120 d, although the PCL and PCL/ES groups had smaller numbers of positive cells (70 cells), there was no statistical difference among the groups.

While the individual evaluation of positive RANKL and OPG cells in each group is an interesting measurement, the RANKL/OPG ratio (quotient between both positive markings) can provide a better interpretation of the tissue remodeling process, as shown in Figure 6c. Ratio values greater than 1 suggest a trend towards bone resorption/degradation, as RANKL (binding protein) interacts with the RANK receptor in the plasma membrane of osteoclasts for cell activation and remodeling stimulation. Values less than 1 suggest a trend of bone formation, as OPG inhibits RANKL from binding to RANK (antagonist), and values close to 1 suggest a balance between both stimuli. After 30 d, the untreated, ES, PCL, TCP, and TCP/ES groups presented stimulus for bone resorption with a ratio of approximately 1.6 for the untreated group, while the others had an approximate ratio of 1.2 to 1.4. The HA and HA/ES groups presented ratios close to 1 (above 0.8). After 60 d, the untreated group continued to show the highest ratio (1.6). HA and TCP/ES were close to 1.2, while the other group’s ratios were close to 1. After 120 d, the untreated PCL, PCL/ES, and HA groups presented ratios above 1.2, while the ES and HA/ES groups were close to 0.8, and TCP and TCP/ES were close to balance (ratios below 1.2).

## 4. Discussion

Failure rates in the resolution and reconstruction of critical bone defects have encouraged the development of scaffolds with material properties close to natural tissues with the help of tissue engineering. Investigations concerning complementary therapies for promoting cell activity and enhancing the cell–biomaterial interaction, have also been encouraged [24,25].

The use of polymer-ceramic composite scaffolds is promising for the treatment of large bone defects. It is particularly relevant to use additive manufacturing which allows the production of 3D porous, biodegradable, and biocompatible scaffolds. Moreover, bone healing can also be enhanced by the application of non-invasive electrical stimulation (capacitive coupling) at physiological and therapeutic levels. The present study investigated the use of polymeric PCL/β-TCP (20 wt%) and PCL/HA (20 wt%) scaffolds, in combination with non-invasive electrical stimulation for bone regeneration. We used an extrusion-based additive manufacturing system to successfully produce well-defined 3D scaffolds with pore size, filament diameters, and porosities similar to those of the computer-aided designs previously reported by Huang et al. [7]. PCL/HA scaffolds show greater biocompatibility and cell proliferation than PCL/TCP scaffolds, while PCL/TCP scaffolds have better mechanical properties than PCL/HA scaffolds. Moreover, the nano-scale HA showed better mineralization than micro-scale TCP particles, acting as a nucleate site.

Lohfeld et al. [8] evaluated the use of PCL scaffolds manufactured with different concentrations of β-TCP (10% to 50%), along with the selective laser sintering (SLS), technique to treat the tibia bone of sheep. The authors reported that a concentration of 10% β-TCP showed limited results when compared to other concentrations. However, as proposed by Huang et al. [7], β-TCP (and HA) concentrations above 30% could compromise the mechanical strength of the scaffolds produced by screw-assisted additive manufacturing systems due to the fragility of ceramic materials. For this reason, 20 wt% was used for each ceramic biomaterial in this study.

Although bone is capable of healing by itself after tissue damage, large bone defects are compromised. A critical-sized bone defect in the calvary of rats has been reported by several authors as the smallest defect that does not present spontaneous regeneration over time without treatment. Spontaneous tissue regeneration does not occur when there is no intervention [26,27]. Considering the results presented in this research paper, the bone defect treated with scaffolds presented mineralized tissue formation over the experimental period with greater prevalence in the polymer-ceramic composite groups (with HA and TCP scaffolds), indicating that the treatments used in the experimental model were satisfactory. This was also supported by the gene expression data.

Although we did not assess inflammatory or immune responses, no signs of infection or pronounced inflammation were observed in any animal over the experimental period. This is supported in a review by Brunello et al. [28], in which there were no reported adverse reactions to the implanted ceramic composite materials in animal models. Critical-sized calvary bone defects were used in the vast majority of studies to evaluate the osteogenic potential of ceramic composites in rats, the same model we used in our investigation.

Initially, histological and histomorphometric analyses were performed. Although no significant differences were found among the groups in the number of blood vessels (angiogenesis), the vascular area was greater in the HA/ES group at both 30 and 60 d, possibly due to the osteoconductive properties of the scaffold material and the electrical stimulus which improved cell adhesion, proliferation, and blood vessel formation. Therefore, it can be inferred that there was an increase in vascularization due to the greater diameter of the vessels, especially in the initial phase, where vascularization plays an important role in bone formation. These data agree with the literature [2,26,29] in pointing to the angiogenic potential of the ES at 10 μA intensity, since the use of scaffolds without ES were not as effective.

In addition, after 120 d, the use of polymer-composite scaffolds and ES (HA, HA/ES, TCP, and TCP/ES groups) presented fewer blood vessels than the other groups; however, a greater vascular area could be seen, mainly in the ES groups (HA/ES and TCP/ES). As bone healing progressed, the number of blood vessels tended to decrease once the primary bone tissue is formed. The histological data corroborated the gene expression evaluation, as greater stimulus to osteogenesis was also related to a decrease in the number of blood vessels later in the healing process. However, fewer vessels and larger vascular areas were reported, supporting osteogenesis, even in more advanced stages. The grafting in groups without ES treatment was not as effective when compared to the groups with scaffolds and ES (PCL/ES, HA/ES, and TCP/ES). This, along with the histomorphometric data (number of vessels and vascular area formed by vessels) and Vegf gene expression, point to the angiogenic potential of ES therapy.

Moreover, the in vivo findings reported by Leppik et al. [18], for bone defects treated with ES, showed a reduction in the number of blood vessels, with the consequent reduction of fibrous tissue and an important increase in mineralized tissue after 4 weeks. Similar results were reported in the present study. The polymer-ceramic composite scaffold groups presented a lower number of blood vessels compared to the PCL, PCL/ES, ES, and untreated groups over the period, but showed greater mineralization. The prevalence of connective tissue instead of mineralization is a classic characteristic of clinical cases of non-bone union. The data are also supported by our prior studies of osteogenesis, scaffolds, and electrical current therapy in an animal model of critical bone defect repair [26,29].

Bins-Ely et al. [30] used ES (10 μA and 20 μA intensities) directly on titanium implants in the tibia of dogs to assess osteointegration and bone formation. The authors reported that the application of 20 μA promoted greater bone area formation between the bone tissue and the implant after 15 d. Although the experimental animal model and ES approach were different (the authors used the direct ES), it was noteworthy that the low intensity ES used also favored osteogenesis.

Zhang et al. [31] reported the in vivo use of HA and β-TCP in three-dimensional printed polylactic acid scaffolds. By histological evaluation, the use of HA scaffolds was reported showing interesting data after 30 d of experiment; however, the authors reported greater bone volume in defects filled with β-TCP after 60 d, possibly due to its osteoinductive properties over longer experimental periods. As reported, and observed in our work, promising results were initially reported with HA scaffolds in mineralization and vascular areas, while β-TCP scaffolds showed more effective performance after 60 d. In addition, the data corroborate the in vitro findings of Jin and Kim [32], who demonstrated that PCL/β-TCP scaffolds associated with ES showed greater mineralized tissue formation when compared to scaffolds without β-TCP and ES.

To provide more data regarding the role of polymer-ceramic composite scaffolds and ES for bone regeneration, molecular analysis was performed using genes related to osteogenesis. Runx-2 is essential for the proliferation of osteoprogenitor cells and the differentiation of osteoblasts. As a downstream gene of Runx-2, Osterix is a transcription factor expressed by osteoblastic precursor cells in the perichondrium, which is essential for the differentiation of osteoblasts and positively regulates Vegf expression by binding to Vegf promoters, thereby controlling osteogenesis [32,33]. The increased expression of Runx-2 in the TCP/ES group, and Osterix in TCP and TCP/ES groups, is thought to be related to the osteoinductive potential of β-TCP combined with electrical stimulation, which leads to cell differentiation and ossification. This supports the greater mineralization observed in the histological evaluation and explains the positive contribution to bone remodeling.

Bmp-2 and Bmp-7 are considered osteoinductive bone growth factors that are directly associated with bone progenitor cells, interacting with their respective receptors, and inducing the differentiation of osteoblasts, which leads to bone formation. Moreover, Bmp-7 also contributes to angiogenesis [34,35,36]. The increased gene expression of Bmp-2 in HA, TCP, and TCP/ES groups after 30 d, and in the TCP/ES group after 60 d, suggests that osteoprogenitor cells had greater stimulus for tissue formation and mineralization. As reported by Fei et al. (2012) [37], β-TCP associated with calcium salicylate stimulated the gene expression of Bmp-2 and Runx-2, controlling the proliferation and differentiation of cells of the osteogenic lineage, as also shown in our study, supported by histological evaluation. It is evident that the association of β-TCP with PCL and ES enhanced mineralization during the bone regeneration process.

In addition to Runx-2, Osterix, Bmp-2, and Bmp-7, osteopontin, one of the main non-collagen proteins produced at the end of the mineralization process, showed higher expression in the TCP group after 30 d and in the TCP/ES group after 60 d. This could provide a well-organized extracellular matrix, coordinating matrix–cell and matrix–mineral interactions, corroborating with the higher mineralized tissue. Moreover, OPN is considered to play an important role in bone formation and resorption. It is highly concentrated in newly formed bone and at bone surfaces, and it has been demonstrated that OPN has chemotactic activity on the precursor of osteoclasts [38,39]. Corroborating our data, the TCP/ES presented a higher mineralized tissue area after 60 d, with large portions of mature bone due to the bone remodeling phase, which strongly suggests that it is a promising bone substitute.

The gene expression results justify the higher percentage of osteoid and mineralized tissue in HA and HA/ES groups initially (30 d) and TCP and TCP/ES after 60 d. These results corroborate the literature regarding the use of scaffolds serving as a framework to favor migration, proliferation, cell differentiation, and consequently, tissue formation [29,40,41]. Despite significant advances in the application of calcium phosphates as osteoinductors, their interaction with stem cells and the bone defect portion have not been completely elucidated. The hypothesis that microarchitectural features act as key drivers for calcium phosphate-led osteogenesis has gained importance over the past decade. In addition, free ions, specifically calcium, possibly released from these materials to the surrounding environment, also showed the ability to induce osteogenesis in mesenchymal stem cells through the stimulation of Bmp-2 expression [38,41], and was observed in our in vivo results.

As already demonstrated by our research group, in vivo models, regarding the use of ES at 10 μA for 5 min twice a week [21,26,29], are supported by the literature on in vitro models [20,42], regarding the conclusion that the use of ES has an important influence on modulating the bone remodeling phase. A balance in the RANKL/OPG signaling system is essential for bone homeostasis. Under homeostatic conditions, the expression levels of RANKL and OPG were balanced. The relative ratio of RANKL/OPG controls osteoclast differentiation, which plays a central role in regulating bone remodeling. The results provide evidence on how the application of ES modulates the RANKL/OPG system favoring tissue formation, because the untreated group showed ratios greater than 1 over the experimental period, while the ES group presented ratios less than 1 after 60 d.

However, when using scaffolds as bone substitutes to support bone tissue formation, the same findings were not observed, as a support matrix was used for cell influx, adhesion, and proliferation. Although the ratios of PCL and PCL/ES groups were close to those of the polymer-ceramic groups, the mineralized tissue in these last groups was much higher at 30 and 60 d, demonstrating the positive effect of the polymer-ceramic composite scaffold over only PCL. The mineralized tissue formed early after 30 d in both HA groups and can be justified by the osteoconductive properties of the ceramic constituent, which provide greater cellularity and consequently, as shown, the appropriate gene expression of Bmps and a RANKL/OPG ratio less than 1, resulting in faster tissue formation.

However, both TCP groups showed greater formation of mineralized tissue after 60 d in addition to higher expression of osteogenic genes, possibly due to the osteoinductive properties of TCP, as confirmed by histological data. After 120 d, although all scaffold-treated groups presented greater mineralized tissue, the data suggested that the polymer-ceramic composite scaffold groups present a greater volume of mature tissue. The tissue formation was faster and therefore, in an advanced remodeling phase. In this same period, both groups with PCL scaffolds had higher ratios, while the TCP groups seemed to be in homeostatic equilibrium.

The use of non-invasive therapies that activate signaling pathways and/or cellular interactions can have positive effects on tissue engineering. The combination of ES and bone tissue engineering has the potential to create synergy that can provide results that far exceed those achieved by any independent treatment [26,43]. The data presented suggest positive impacts of the use of polymer-ceramic composite scaffolds when stimulated by ES at 10 μA for 5 min twice a week, favoring the replacement of connective tissue by mineralized tissue (mineralization process) and its development into mature bone (bone remodeling process). More research is needed at the molecular level to understand the cell signaling pathways associated with ES.

## 5. Conclusions

This paper presented an in vivo study of 3D printed polymer-ceramic composite scaffolds. The use of PCL associated with β-TCP 20 wt% and PCL associated with HA 20 wt% presented evidence of a positive impact for bone tissue engineering. The use of β-TCP 20 wt% scaffolds provided strong evidence of enhanced long-term application with regard to the bone regenerative process of critical-sized bone defects when compared to the PCL/HA 20 wt% scaffolds. Moreover, the use of electrical stimulation as a non-invasive and complementary therapy boosted the bone regeneration effect of PCL/β-TCP scaffolds providing a two to three-fold change in angiogenic and osteogenic gene expression, resulting in greater mineralized tissue formation after 60 d. In addition to osteogenesis, PCL with β-TCP composite scaffolds and the ES also modulated the bone remodeling, providing the expected balance between formation (early stages, 30 d to 60 d) and maturation (later stages, 60 d to 120 d) during the RANKL/OPG physiological process.

## Figures and Tables

**Table 1:** Experimental groups for in vivo study.

Group Number	Group Name	Scaffold Implanted	Electrical Stimulation	PCL Polymer Concentration	Ceramic Concentration
1	Untreated	No	No	0 wt%	0 wt%
2	ES	No	Yes	0 wt%	0 wt%
3	PCL	Yes	No	100 wt%	0 wt%
4	PCL/ES	Yes	Yes	100 wt%	0 wt%
5	HA	Yes	No	80 wt%	HA 20 wt%
6	HA/ES	Yes	Yes	80 wt%	HA 20 wt%
7	TCP	Yes	No	80 wt%	β-TCP 20 wt%
8	TCP/ES	Yes	Yes	80 wt%	β-TCP 20 wt%

## Data Availability

Data available on request from the corresponding author due to privacy and also ethical issues.
